# Circle of Security—Parenting and Parent–Child Interaction Therapy—Toddler: A Qualitative Exploration of Parents' Perspectives

**DOI:** 10.1111/sjop.70009

**Published:** 2025-07-23

**Authors:** Jane Kohlhoff, Sara Cibralic, Nancy Wallace, Susan Morgan, Linda Lennie, Lucinda Rabbetts

**Affiliations:** ^1^ Discipline of Psychiatry & Mental Health, School of Clinical Medicine University of New South Wales Sydney New South Wales Australia; ^2^ Karitane Sydney New South Wales Australia

**Keywords:** attachment‐based interventions, circle of security‐parenting, disruptive child behaviors, parent–child interaction therapy—toddler, parenting

## Abstract

Parent–Child Interaction Therapy—Toddler (PCIT‐T) and Circle of Security—Parenting (COS‐P) are two attachment‐based early parenting programs with emerging evidence bases. Most of the research has, however, been quantitative in nature. Understanding caregiver perspectives and acceptability of the programs is therefore needed. This study aimed to address this gap in research by examining perspectives of parents who participated in PCIT‐T or COS‐P at an Australian community‐based child behavior clinic for treatment of toddler behavior problems. Twenty‐nine mothers were purposively recruited to participate in a semi‐structured post‐program interview (COS‐P: *n* = 10; PCIT‐T: *n* = 19). Data were analyzed using an inductive thematic analysis approach. Results of thematic analysis showed that parents in both groups experienced a range of positive gains (for the toddler, themselves as a parent, and for relationships). Participants in both groups identified the clinician as a key facilitator of positive program outcomes, and time commitment as a barrier. For the COS‐P group, the group process and treatment journey were identified as facilitators, and inconsistent attendance from group attendees was a barrier. The PCIT‐T group viewed the live coaching and the manualized protocol as facilitators. Results suggest that both COS‐P and PCIT‐T are viewed positively by parents and identify several parent‐identified facilitators/barriers.

**Summary**

- Parent–Child Interaction Therapy—Toddler (PCIT‐T) and Circle of Security—Parenting (COS‐P) are popular attachment‐based early parenting programs with emerging evidence bases.
- Using a qualitative methodology, parents' perspectives of PCIT‐T and COS‐P were evaluated.
- Results provide insight into the acceptability, facilitators, and barriers of positive treatment outcomes.

## 1 Introduction

Approximately, 1 in 7 children experience a mental health condition (Abel et al. 2019; Georgiades et al. 2019; Lawrence et al. 2015; Whitney and Peterson 2019) and in many cases, this represents the start of a trajectory towards lifelong mental health issues (Kim‐Cohen et al. 2003). One of the strongest predictors of childhood mental health is the quality of the early caregiving environment (Scully et al. 2020), with the first 2 years of life known to be of particular salience given high levels of neuroplasticity and sensitivity to environmental influences (Moore et al. 2017). Specifically, evidence suggests that caregivers who are sensitive and contingently responsive to their infant/young toddler's emotional needs provide a nurturing environment in which core social, emotional, and cognitive capacities develop and flourish (Lyons‐Ruth 1996). In contrast, for infants/toddlers who experience adverse early caregiving (i.e., non‐sensitive, harsh, hostile, or inconsistent parenting), key social and emotional developmental processes can be disrupted. This can have a cascading effect on the child's social–emotional development—often manifesting initially in the toddler years as behavioral problems (e.g., externalizing or internalizing problems) and then in later childhood and adolescence as mental health conditions (Lomanowska et al. 2017).

Given the impacts of early parenting quality on later child mental health outcomes, the value of delivering parenting interventions to at‐risk families in the first 2 years of life has been acknowledged (Australian Government National Mental Health Commission 2021; Australian Government Productivity Commission 2020; NSW Ministry of Health 2019). Numerous ‘attachment‐based’ parenting programs aimed at increasing sensitive caregiving and decreasing harsh or non‐attuned parenting in the early years have been developed, and many of these have been shown to be associated with positive changes in both parenting quality (e.g., enhanced sensitivity) and child outcomes (e.g., increased rates of attachment security and reductions in externalizing behavior) (Bakermans‐Kranenburg et al. 2003, 2005; Facompré et al. 2018; Kohlhoff and Cibralic 2021; Letourneau et al. 2015; Steele and Steele 2018; Van Ijzendoorn et al. 1995; Wright et al. 2015, 2017; Wright and Edginton 2016).

One of the programs that is popular in Australia is Circle of Security—Parenting (COS‐P; Cooper et al. 2009), an 8‐week parent education group program designed for parents of children aged 0 to 12 years. COS‐P was developed as an adaptation of the original 20‐week Circle of Security—Intensive (COS‐I) program (Powell et al. 2014). The COS programs are theoretically underpinned by the Attachment Theory (Bowlby 1988). The theory, which was originally proposed by Bowlby (Bowlby 1946, 1969), and further refined through the work of Ainsworth (Ainsworth et al. 1978), posits that through early experiences with their primary caregivers, infants develop expectations about their relationship with their caregivers (i.e., internal working models). These expectations inform their attachment styles. Secure attachment styles emerge as a result of sensitive and consistent caregiving, while insecure‐avoidant and insecure‐resistant attachment styles have been linked to distant and inconsistent caregiving, respectively. Conversely, children with a disorganized attachment style have caregivers who are frightened and/or frightening (Granqvist et al. 2017; Schuengel et al. 1999).

Like the COS‐Intensive program, COS‐P aims to improve child attachment security by increasing caregiver sensitivity and responsiveness to child cues, parental reflective functioning, parental emotion regulation, parental representations of the child, and parental awareness of the impact of their personal attachment history on their caregiving patterns (Cooper et al. 2009). The COS‐P group program was designed for delivery across eight, 2‐h group‐based sessions, led by facilitators who have been trained and certified by COS‐International. Each session involves the presentation of pre‐recorded videos, explanation of key concepts through engaging graphics and simple language, and group discussion. The program uses the ‘COS graphic’, an engaging picture that shows the child's need for exploration from a secure base (top of the circle) and comfort (bottom of the circle), and the parent's role in supporting the child's movement around the circle (represented with two hands representing the parent's role in providing the child with a ‘secure base’ when exploring and a ‘safe haven’ or comforter in times of distress). Throughout the COS‐P program, parents are also encouraged to reflect on their own early parenting experiences and emotional triggers (introduced with the metaphor of ‘shark music’), and to be aware of times that their child ‘miscues’ (acts in ways that protect him/her from the pain or discomfort of having a healthy need exposed or unmet).

There is a growing evidence‐base to support the clinical effectiveness of COS‐P. Utilizing a randomized control trial (RCT) design, Cassidy et al. (2017) compared outcomes of COS‐P with a waitlist control condition in a sample of 141 mother–child dyads (child age range = 40.45–63.58 months; *M* = 50.68 months, SD = 5.94) from low socioeconomic communities in the United States. Results showed that while there were no treatment effects on child attachment security or child externalizing behavior, COS‐P was associated with a significant reduction in mothers' unsupportive responses to their child's distress. In a Swedish study, Risholm Mothander et al. (2018) compared the effects of COS‐P and a non‐treated control condition on parents' internal representations and the quality of parent–child interactions. The sample comprised 52 parent‐infant dyads (children aged 0–4 years) recruited from infant mental health clinics. Results showed that while there were no significant differences between groups at follow‐up in terms of the quality of caregiver‐child interactions, parents who attended a COS‐P group displayed more positive representations of their children, and of themselves as caregivers. Most recently, Zimmer‐Gembeck et al. (2022) conducted a RCT with 85 caregiver‐child dyads (COS‐P *n* = 51, waitlist controls *n* = 34; child age 1 to 7 years) referred for child behavior and/or parenting problems. Results showed the COS‐P intervention to be associated with greater declines in caregiver attachment anxiety. However, there were no significant post‐treatment differences between groups in terms of child behavior (externalizing or internalizing), caregiver stress, reflective functioning, positive parenting practices, attachment avoidance, or depressive symptoms.

Parent–Child Interaction Therapy—Toddlers (PCIT‐T; Girard et al. 2018) is another parenting program that has a growing evidence‐base. Developed as an adaptation of standard PCIT (Eyberg and Funderburk 2011), PCIT‐T incorporates elements from both attachment theory and social‐learning theory. Attachment theory principles (i.e., parent as the child's safe haven in times of distress) are drawn on in the CARES techniques (CARES stands for: **C**ome in; **A**ssist child; **R**eassure child; **E**motional validation; and **S**oothe) a set of techniques that parents are encouraged to use in response to their child's negative emotions (Girard et al. 2018). In addition to using the CARES techniques to support their child's emotion regulation, parents are also encouraged to apply a corresponding set of ‘adult CARES’ techniques to support their own emotional regulation (Girard et al. 2018).

Social learning theory is an alternative theory for understanding how parent–child interactions impact child development; it suggests that both direct and indirect experiences shape behaviors (Bandura 1971). With the primary sources of these exposures and experiences in the early years being the parent–child and family environment, parenting behavior is thought to be the most significant influence on child behavior. Within the Social Learning Theory framework, it is thought that child behavior can be improved through parental incorporation of positive attention and praise for desirable behavior as well as establishing clear boundaries for undesirable behavior (Patterson 1982). Through the incorporation of social learning theory principles (i.e., positive attention for desired behavior and establishment of boundaries for undesired behavior), PCIT‐T aims to improve basic parenting skills in order to promote positive child behavior (Girard et al. 2018).

The PCIT‐T intervention comprises two sequential treatment phases, Child‐Directed Interaction‐Toddler (CDI‐T) and Parent‐Directed Interaction‐Toddler (PDI‐T). Both phases center around parent–child play and involve parent education and live parent coaching through a one‐way mirror (if clinic‐based) or through video conferencing (if conducted via the internet). The CDI‐T phase aims to (1) improve positive parenting skills (e.g., increase use of labeled praises, reflections, and behavioral descriptions), (2) reduce the use of negative parenting behaviors (e.g., reduce use of critical statements, questions, and commands), and (3) increase parental awareness of, and sensitivity to, their child's emotions, and to actively support the child's emotion regulation during times of distress. Throughout the CDI‐T phase, there is also a focus on enhancing the parent's own emotion regulation abilities and increasing their reflectiveness with regards to the connection between their own emotion regulation and behavior and that of their child. The PDI‐T phase aims to enhance toddler compliance/listening skills by using a fun, game‐like sequence in which parents are supported to use direct, developmentally appropriate commands and the child receives positive reinforcement for listening. Sessions are typically weekly or twice weekly, and graduation from the program occurs once families have successfully been able to use the CDI‐T and PDI‐T skills (Girard et al. 2018).

To date, two RCTs have been conducted to test outcomes of PCIT‐T (Kohlhoff et al. 2021; Kohlhoff et al. 2024). In the first study (Kohlhoff et al. 2021), 66 mother‐toddler dyads (*M* child age = 19.13 months, range = 15–24) referred to a community‐based child behavior clinic for treatment of behavioral problems were randomly allocated to receive PCIT‐T (CDI‐T phase only) or to be in a non‐treated waitlist condition. Results showed that PCIT‐T led to a greater degree of improvement in parenting quality (increased positive parenting skills and sensitivity, and decreased intrusiveness) and child outcomes (reduced externalizing and internalizing behaviors). A follow‐up study showed that these PCIT‐T treatment outcomes were maintained up to 4 months post‐treatment (Kohlhoff, Morgan, Briggs, et al. 2020). The second study, undertaken by the same research team (Kohlhoff et al. 2024) and the study from which the sample reported in this paper was drawn from, included 90 caregiver‐child dyads (*M* child age = 19.48 months, range = 14–24) referred to a community‐based child behavior clinic. Participants were allocated to receive either PCIT‐T (*n* = 30; *M* child age = 19.31, SD = 3.38), COS‐P (*n* = 30, *M* child age = 19.12, SD = 3.10) or to be in a non‐treated waitlist condition (*n* = 30, *M* child age = 20.08, SD 2.95). Results showed that compared to COS‐P and waitlist control groups, PCIT‐T participants displayed significantly greater improvements in parenting quality (increased sensitivity and parenting skills) and child outcomes (improved social competence and reduced internalizing problems and general behavior issues). Of note is that COS‐P participants also showed significant increases in parenting quality and decreases in parental stress and child externalizing behavior.

While quantitative studies provide vital information about the effectiveness of attachment‐based early parenting interventions in key intervention target areas (e.g., parenting sensitivity, child behavior), qualitative studies can provide useful additional information about program effectiveness, mechanisms of change, and facilitators and barriers to program success (Rich and Ginsburg 1999). Information in these areas can be instrumental in facilitating positive ‘real world’ implementation outcomes. Importantly, the results of qualitative studies can help clinical service providers to make informed decisions about which programs to offer, and to whom, and ways in which outcomes can be maximized. To our knowledge, there have been two qualitative studies exploring parents' perspectives about COS‐P (Helle et al. 2023; Maxwell et al. 2021) and one study exploring parents' perspectives about the PCIT‐T program (Kohlhoff, Cibralic, and Morgan 2020).

In the Maxwell et al. (2021) study, interviews and focus groups were conducted with 14 parents who had attended a COS‐P group and 20 COS‐P facilitators; transcripts were analyzed using an inductive thematic analysis approach. The researchers who conducted the interviews and analysis were not involved in the facilitation of the participant COS‐P groups. Results suggested that COS‐P brings change for parents and children by altering the ‘lens’ through which parents view the child, themselves as a parent, and the parent–child relationship. Participants in this study spoke about the way that this new lens led to new levels of empathy, compassion, and parenting confidence. The study also highlighted four underpinning components of program impact (key content, skills practice, group processes, and support from the facilitator) and identified program limitations including reduced effectiveness for parents from non‐English speaking backgrounds, with limited intellectual capacity or poor reflective capacity, or for parents who were for whatever reason not yet ready to work on their relationship with their child. The study had strengths including the fact that each of the parents in the sample had attended one of 13 different COS‐P groups delivered by seven different facilitators, thus reducing the risk that positive evaluations were related to individual groups or facilitators. The major study limitation was related to the sample composition: the participant response rate was not reported, and the authors pointed out that feedback about the program was overwhelmingly positive and so it is likely that the participants who volunteered for the study did so because they felt positively about it, highlighting a potential source of bias. Another limitation was related to coding reliability. While the author's use of an iterative consultative process to develop and refine the themes was a study strength, the absence of inter‐coder reliability checks to confirm the validity of the themes means that biases may still have been at play. In the Helle et al. (2023) study, interviews were undertaken with 12 female caregivers diagnosed with a mental health condition who participated in a COS‐P at a district psychiatric health centre in Western Norway. Transcripts were analyzed using reflexive thematic analysis. The researchers who conducted the interviews and analysis did not know the interviewees and had not been involved in the facilitation of the COS‐P groups. Results indicated that COS‐P allowed participants to gain insights into their own developmental histories and increased their self‐awareness and their sense of competence as mothers. A strength of this study is that it was undertaken in a clinical environment, enhancing the generalisability of findings to clinical settings. The study was limited by the failure to report participants response rates as well as limited participant information (e.g., number of children, family situation, and treatment history), making it difficult to form a coherent picture regarding participants.

In the Kohlhoff, Morgan, Briggs, et al. (2020) study, interviews were conducted with five parents who completed the CDI‐T phase of PCIT‐T; transcripts were analyzed using an inductive thematic analysis approach. The researchers who conducted the interviews and analysis were not involved in the facilitation of the PCIT‐T intervention for participants in the study. These parents talked about having learned new parenting strategies, increasing their confidence as a parent, and noticing improvements in the quality of their relationship with their child. The study also identified several program components that led to these changes, namely the live coaching, the relationship with the therapist, and the home‐based practice of skills between sessions. While this study had strengths (e.g., conducted in a real‐world clinical setting), it was limited by the fact that it only evaluated the CDI‐T phase of the program, and similar to the Maxwell et al. study, the authors did not apply any inter‐coder reliability checks to confirm the validity of the identified themes.

Given the lack of qualitative evaluations of the COS‐P and PCIT‐T programs, the aims of the current study were thus to use a qualitative methodology to (1) explore and contrast the perspectives of two groups of parents (who presented with concerns about toddler behavior) regarding the acceptability and perceived impacts of the COS‐P and PCIT‐T programs; and (2) identify factors that facilitated or presented barriers to the successful implementation and outcomes of the two interventions. While the two programs are distinct and not perfect comparators, the decision was made to analyze the programs together as (1) both are attachment‐based programs; (2) both programs contained a similar cohort of participants (i.e., parents of toddler‐aged children with challenging behaviors seeking intervention); and (3) both programs have a similar ‘dose’ (16 h across 8 weeks). The overall goal of this work was to obtain information that could be used to refine the delivery of the COS‐P and PCIT‐T programs and assist clinicians and clinical services with decision‐making around choice of programs based on consumer feedback and experiences, with an ultimate goal of enhancing clinical outcomes for families.

## 2 Method

### 2.1 Study Participants

Demographic characteristics of the sample are shown in Table 1. As shown, participants were 29 mothers ranging in age from 25–45 years (*M* = 34.66, SD = 5.18) with children aged 14 to 24 months (*M* = 18.58, SD = 3.27). All participants took part in a larger randomized controlled trial (RCT) study testing outcomes of the PCIT‐T and COS‐P parenting interventions (Kohlhoff et al. 2024). As part of the study protocol, participants were invited to participate in a qualitative interview within 1–3 weeks following the completion of their allocated intervention condition (PCIT‐T or COS‐P). In total, of the 60 participants allocated to COS‐P or PCIT‐T, 37 participants completed the intervention. Of these, 29 participated in a qualitative interview (*n* = 10 who took part in COS‐P; *n* = 19 who took part in PCIT‐T). The lower number of COS‐P participants reflects the greater participant attrition observed in the COS‐P group (Kohlhoff et al. 2024). Compared to parents from the larger RCT who did not participate in a qualitative interview, those who participated tended to be older, *T* = −2.15 (50), *p* < 0.05. There were no other significant differences between those who did and those who did not participate in an interview in terms of mean child age, marital/relationship status (single versus partnered), education level (university versus not university educated), ethnicity (Caucasian versus non‐Caucasian), or child behavior severity at admission (assessed using the Child Behavior Checklist Externalizing Subscale Score, (Achenbach and Rescorla 2000)) (*p*'s > 0.05).

**TABLE 1:** Sample demographics.

Variable	COS‐P (*n* = 10)	PCIT‐T (*n* = 19)
Mother age in years (Mean ± SD, range)	34.4 ± 4.7, 27–42	34.8 ± 5.6, 25–45
Child age in months (Mean, SD, range)	18.1 ± 3.8, 15–24	19.0 ± 3.03, 15–23
Relationship status		
Partnered (*n*, %)	6, 60	3, 15.8
Single (*n*, %)	3, 30	16, 84.2
Language spoken at home		
English (*n*, %)	9, 90	12, 63.2
Arabic (*n*, %)	0	2, 10.5
Cantonese (*n*, %)	1, 10	1, 5.1
Farsi (*n*, %)	0	1, 5.1
Spanish (*n*, %)	0	1, 5.1
Vietnamese (*n*, %)	0	1, 5.1
Ethnicity		
Caucasian (*n*, %)	7, 70	4, 21.1
Asian (*n*, %)	1, 10	4, 21.1
Hispanic (*n*, %)	1, 10	1, 5.1
Middle Eastern (*n*, %)	1, 10	3, 15.8
Aboriginal or Torres Strait Islander (*n*, %)	0	1, 5.1
European (*n*, %)	0	3, 15.8
Did not specify (*n*, %)	0	3, 15.8

Participants in the COS‐P group ranged in age from 27 to 42 years (*M* = 34.4 years, SD = 4.71), and their children ranged in age from 15 to 24 months (*M* = 18.11, SD = 3.82) at the time of recruitment into the study. Sixty percent were married, 70% identified as Caucasian, and 90% indicated that the primary language spoken at home was English. Participants in the PCIT‐T group ranged in age from 25 to 45 years (*M* = 34.82, SD = 5.58) and their children ranged in age from 15 to 23 months (*M* = 19.0, SD = 3.03) at the time of recruitment into the study. Fifteen percent were married, 21% identified as Caucasian, and 63% indicated that the primary language spoken at home was English.

### 2.2 Procedure

This study was conducted at a community‐based child behavior treatment clinic located in the South‐Western region of metropolitan Sydney, Australia. The study was positioned within a larger RCT assessing the efficacy of PCIT‐T and COS‐P on child behavior (Kohlhoff, Cibralic, Wallace, et al. 2020). Families with children aged 14 to 24 months who were referred to the clinic by their primary health care provider were informed about the RCT during a telephone intake. Families were eligible to participate if they indicated that they had concerns about their child's behavior and/or had difficulty managing their child's behavior; the participating parent had English‐speaking and English‐writing proficiency; and the participating parent did not have an intellectual disability or psychiatric condition that may have impacted their ability to participate in, or complete, the treatment and/or assessment measures. Informed, written consent was provided by participants during their initial face‐to‐face appointment at the clinic. In total, 90 families participated in the larger RCT, with 30 mothers in COS‐P, 30 in PCIT‐T, and 30 in a waitlist control condition. All families who completed COS‐P or PCIT‐T treatments in the larger RCT were invited to take part in the qualitative component of the research. Interviews were continued even after saturation was reached (i.e., interviews were no longer producing new insights) to give all participants the opportunity to participate in the qualitative interview and to validate the consistency of themes across interviews (Guest et al. 2006).

Interviews were conducted by research assistants, who were not involved in the delivery of the COS‐P or PCIT‐T clinical interventions, within a week of participants completing treatment. All research assistants were female, aged between 20–35 years, with a background in psychology and experience undertaking qualitative research. One‐on‐one, in‐person interviews were conducted with all participants. A semi‐structured interview schedule format was used as it permitted for consistent questioning across areas of interest (e.g., positives and/or negatives of the intervention), while also allowing for other questions that arose during the interview in response to participants' unique experiences. The interview questions were developed by authors based on clinical and research experience. Examples of questions asked included: “thinking back to how you were feeling before you started the program, can you tell me a little bit about what you were expecting to get out of the program?”, “do you believe the program helped you and your child and your family?” and “was there anything about the program that you didn't like?”. Interviews range in length from 10 to 45 min (average time = 20.36 min) and took place between December 2018 and June 2021. All interviews were recorded and transcribed verbatim. Transcripts were checked against recordings by a research assistant to ensure they were accurately transcribed. To preserve participant anonymity, identifying information was removed from the transcripts. The extracts that appear in this paper were chosen on the basis that they provide the clearest descriptions of themes relevant to the current study. The transcribed interviews have been edited only to the extent that irrelevant words such as “um” and “like” have been removed and irrelevant sentences have been replaced with ellipses (…).

### 2.3 Intervention

#### 2.3.1 Parent–Child Interaction Therapy (PCIT‐T)

Consistent with the PCIT‐T protocol (Girard et al. 2018), families progressed through both the CDI‐T (1 teaching session +4–6 treatment sessions) and PDI‐T (1 teaching session + 2–4 coaching sessions) phases of the intervention. PCIT‐T was delivered to families through in‐clinic coaching from behind a one‐way mirror using a ‘bug‐in‐the‐ear’ device. To maintain treatment fidelity, clinicians completed fidelity checklists at the end of each treatment session and engaged in fortnightly reflective supervision sessions. While the aim was for all families to attend two 45‐min sessions weekly, across 8 weeks, for the 24 parents who completed treatment, the mean total hours of therapy was 13.95 h (SD = 1.92, range = 9.08–16.42) over an average of 11.42 weeks (SD = 2.60, range = 7–18).

#### 2.3.2 Circle of Security—Parenting (COSP)

In accordance with the protocol outlined by Cooper et al. (Cooper et al. 2009), COS‐P was delivered in a group setting with the aim of participants receiving 16 h of therapy by attending one 90‐min group therapy session across 8 weeks. The groups were in‐person and co‐facilitated by two clinicians trained and accredited in delivering the intervention. Across the RCT trial, 7 COS‐P groups were run. To support participation, childcare was provided to group participants on site. To ensure treatment was delivered as intended, fidelity checklists were completed by facilitators at the end of each session, and facilitators engaged in two 1 h clinical reflection supervision sessions during each group program. Of the 11 participants who completed the COS‐P intervention, the mean number of sessions attended was 6 (SD = 1.27, range = 4–8) and the mean total amount of group time was 11.72 h (SD = 2.86, range = 8—16).

### 2.4 Analysis

With an interest in exploring the ‘user's experience’, data was analyzed using an inductive thematic analysis approach, applied with a phenomenological and essentialist‐realist theoretical framework (Braun and Clarke 2006). This approach was chosen because it allowed for themes to be identified from the data based on the experiences of participants and the meanings that they attributed to them, without the imposition of pre‐existing or pre‐determined theories or categories. In other words, data was evaluated on a semantic level, with themes identified within the surface meaning of data rather than from inferences about meaning beyond what was said (Braun and Clarke 2006).

**FIGURE 1:**
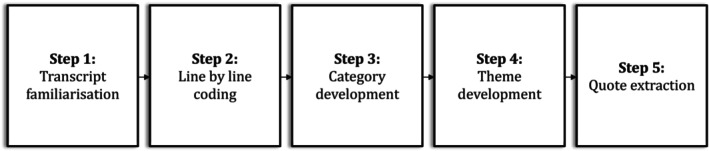
Data analysis steps.

Coding and analysis were undertaken by a team of coders (manuscript co‐authors), allowing for the sharing of viewpoints on data analysis and theory development. In this way, as outlined by Birks et al. (2019), rather than using team members to “check” the quality or “accuracy” of initial codes or the final coding framework, team members engaged in an interactive and iterative process through which they each brought their unique perspectives and together conceptualized and interpreted the data. As shown in Figure 1, this process was conducted across five sequential steps. First, co‐authors read the transcripts independently to familiarize themselves with the data. Second, each individual co‐author examined the transcripts line by line, applying initial codes to each line (e.g., “challenging behavior”, “positive change”, and “gained insight into child behavior”). Third, each individual coder reviewed their initial set of codes and developed overarching categories (themes and sub‐themes) that subsumed most of their initial codes (e.g., program facilitators and barriers, partners). Fourth, the co‐authors then met to share and discuss their individual codes and categories, and through a process of group discussion, refine the categories, identify themes and subthemes (e.g., program outcomes, the group process), develop a coding scheme/code book that articulated these themes and subthemes, and use individual perspectives to reflect individual assumptions and experiences may impact coding. This was particularly important given some of the authors positions in regard the PCIT‐T intervention (i.e., three authors co‐developed the intervention). If coding disagreements arose, they were used as a gateway to reflection and discussion, which aided the reflexive process. Through the process of reflecting and discussing the final thematic structure was reached. Fifth, the transcripts were read again and quotes that best illustrated the themes and subthemes were extracted. To minimize potential coder bias and ensure the inclusion of a range of perspectives, multiple individuals (all co‐authors) were involved in Steps 1–5 described above. Specifically, all transcripts were read and coded by at least two co‐authors and 27% were read and coded by three authors. While all the coders were clinicians at the clinical site at which the study was conducted, none coded transcripts for interventions that they were trained to deliver, or that they delivered for the study.

## 3 Results

**FIGURE 2:**
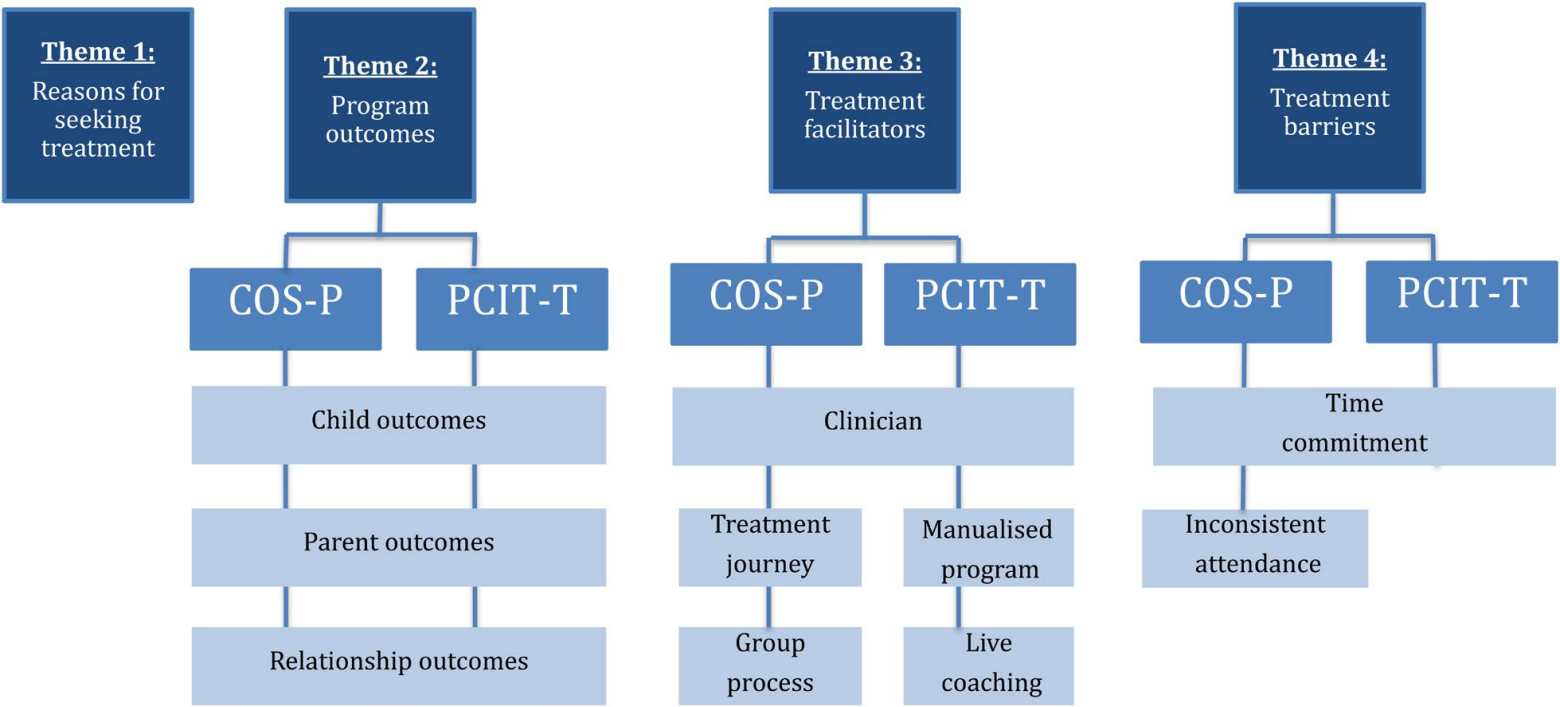
Themes and subthemes.

Four major themes were identified in the data arising from the interviews with the participants in the two intervention groups: (1) reasons for seeking treatment; (2) positive program outcomes; (3) treatment facilitators; and (4) treatment barriers (Figure 2). There were no notable differences between the two intervention groups with respect to the first major theme (reasons for seeking treatment). Within the remaining three major themes, however, 10 sub‐themes were identified, five that applied to both the COS‐P and PCIT‐T interventions and five that were unique to one of the individual interventions. Given that so many of the themes applied to both interventions, results are reported according to theme, with similarities and differences for the two interventions highlighted, as relevant.

### 3.1 Theme 1: Reasons for Seeking Treatment

All participants noted that they sought treatment due to their child's challenging behaviors, with examples including “screaming” (Participants 2, 5, 11, and 15), “tantrums” (Participants 7, 9, 20, 22, 23, and 26), self‐harm (“banging her head on the wall”, Participant 26), and harming others (Participant 13). Some participants reported that they were “struggling” (Participant 8) and feeling “overwhelmed” (Participant 27) and “helpless” (Participant 12); others commented that by attending the intervention program, they were hoping to “learn new skills” (Participant 25) and gain “knowledge” (Participant 28) to help with understanding/managing their child's behavior.

### 3.2 Theme 2: Program Outcomes

While most participants, in both the PCIT‐T and COS‐P groups, noted improvements in their child's outcomes, self‐improvements, and relationship improvements post‐treatment, for others, challenging child behavior continued to be an issue and improvements in relationships were not observed.

#### 3.2.1 Child Outcomes

Participants in both groups noted improvements in child behavior and emotion regulation.

So not only are they (meltdowns) few and far between, but they're shorter… So it's easier to get through it. (Participant 17, PCIT‐T)

My husband can put him to bed now and he'll go happily play and I can leave the house and say, ‘oh I'm going to work now’ without him having a melt down and you know, and he goes to childcare and that's really positive. (Participant 13, COS‐P)

Some participants in the PCIT‐T group also reported improvements in child language.

…his speech also exploded, which was a really nice side outcome. (Participant 21, PCIT‐T)

##### 3.2.1.1 Continued Challenges

Although child behavior improved, some mothers from both the PCIT‐T and COS‐P groups noted that their child continued to experience challenging behaviors. Other mothers, however, reported that they believed the behaviors would resolve over time. One mother stated that while her child's behaviors still exist, her way of reacting to the behaviors has changed.

I was hoping to get sort of more of a directive approach…. I suppose to learn how to manage screaming. For example, what's the best way to manage her not getting her own way? I didn't really get that from the group. (Participant 2, COS‐P)

Well, my son is still having a few challenging issues. But also, I think it just needs to be more time. (Participant 15, COS‐P)

…her misbehavior still exists, but I think she's still … It's mainly, it has changed my view of how to react on her misbehavior… (Participant 16, PCIT‐T)

#### 3.2.2 Parent Outcomes

Mothers from both the PCIT‐T and COS‐P groups also noted increases in their confidence and skills in dealing with their child's behavior. They also gained new insight into why the behaviors were occurring and the impact that their own behaviors were having on their child.

…my self‐esteem is better, my confidence is better, I feel calmer with him. (Participant 9, PCIT‐T)

More confident cause at least I know some tips. Before, nothing…but now I learned more tips [on] how to deal with [her] and how to settle her down…make her closer. (Participant 18, COS‐P)

…before I'd be frustrated and shouting and screaming and now I'm a lot more calm. It's like, you know, ‘How is screaming and shouting going to fix this?’ (Participant 11, COS‐P)

Participants in the COS‐P group reported gaining deeper insight into the ways that their own experiences of being parented were impacting their current parenting style.

I learned about… how things that I had been brought up [with…. had influenced my parenting style in a way that I didn't quite understand before … (Participant 13, COS‐P)

The COS program definitely makes you think about how you were parented, and what you experienced as a child, and your current relationships with your parents and family. (Participant 15, COS‐P)

In contrast, participants in the PCIT‐T group tended to report improvements in their mood and emotional regulation.

My mood is definitely better and [I'm] happier and I enjoy the time I have with her. (Participant 17, PCIT‐T)

I think I'm more in tune with my emotions when I face stressful situations…. I think I'm able to understand his emotions as well. (Participant 10, PCIT‐T)

#### 3.2.3 Relationship Outcomes

Most participants in both groups noted that they saw improvements in their relationships with their child and partner and believed it was due to program attendance.

Child: “I guess our relationship has grown heaps since starting the program. I've bonded with him a lot more… I'm more aware of his emotions and how he's feeling. So, our relationship before was not as deep as now.” (Participant 10, PCIT‐T)

Child: “I think on an emotional level, the bonding is a lot more in‐depth… since the course he will come up to me and give me cuddles and if he's tired, he'll come up and just want snuggling. Whereas prior to that, he just always wanting to be on the go. He didn't want to be held or slowed down.” (Participant 19, COS‐P)

Partner: “… I think we have a more common approach to parenting now, I mean we still have our differences, but this is a very common goal and approach that we both have” (Participant 6, PCIT‐T)

Partner: “I think because I've gone home and explained everything that we've done to him in the sessions and I think before … we both just felt really confused and overwhelmed by [the children]…we've both got different personalities and we'd both been trying different things at the same time and then getting frustrated with each other [be]cause nothing was working so we just blamed each other a lot of the time…whereas now we've been a bit more on the same page and we just have different language now that we can use which is really helpful. And I think it also means they kind of see us as a bit more united front…” (Participant 5, COS‐P)

##### 3.2.3.1 Continued Challenges

The majority of participants in both groups noted improvements in their relationship with their children and partners. Some, however, continued to experience limited change with one COS‐P mother noting that her relationship with her son nor her partner had not changed because of program participation (and choosing not to elaborate further) (Participant 20).

Interviewer So has completing this program impacted your relationship with your child?

Participant 20 (COS‐P) No.

Interviewer And has completing the program impacted your relationship with your partner?

Participant 20 (COS‐P) No.

### 3.3 Theme 3: Treatment Facilitators

Participants from both the COS‐P and PCIT‐T groups discussed a range of factors that enabled positive treatment outcomes. A common theme was that the clinician played a significant positive role; there were also a range of unique factors for each group (PCIT‐T: manualised treatment, one‐way mirror; COS‐P: group process, treatment journey).

#### 3.3.1 Clinician

All participants in the PCIT‐T group spoke of the benefit of having a “support[ice]” (Participant 29), “knowledgeable” (Participant 16), and “passionate” (Participant 24) clinician.

I felt like I wasn't alone, so I wasn't really scared or stressed or nervous when any stressful situations came up. Cause I knew that they would be there to guide me and help me and support me and train me. (Participant 10, PCIT‐T)

Participants in the COS‐P group spoke positively about the group facilitators and acknowledged that they “knew what they were doing” (Participant 13, COS‐P)

The facilitators were really good, they knew what they were doing, they could generate discussions, they addressed questions that came up… (Participant 13, COS‐P)

I found the two ladies that ran the clinic great…they were very reserved and allowed people to speak openly and … they were really good at helping me sort of understand my feelings a bit more … (Participant 1, COS‐P)

##### 3.3.1.1 Unique Themes for the PCIT‐T Group

Participants in the PCIT‐T group consistently identified the treating clinician, the manualised program format, and the live coaching as factors that helped to facilitate positive treatment outcomes.

#### 3.3.2 Manualised Treatment

Participants reported that they thought the program was well‐formatted, and that despite being a manualized program, it that it was “very much personalised” *(Participant 24, PCIT‐T)*.

The signs, the paperwork, the handouts, things that we got, the practicing, that was all really good. (Participant 25, PCIT‐T)

I think I enjoyed everything about the program and how all the sessions were set up, how the rooms are set up with all the toys, it felt very age appropriate. (Participant 10, PCIT‐T)

#### 3.3.3 Live Coaching

Several participants spoke about the benefits of the live coaching from behind the one‐way mirror, but for some it took a couple of sessions to feel comfortable with ‘being watched’.

It was very nice having someone there watching and literally talking to us step by step as things were happening in front of us. (Participant 29, PCIT‐T)

It's good that he can't see and he doesn't get distracted. I can hear, just guiding me how to be a good parent. (Participant 4, PCIT‐T)

At the start it's weird because you know somebody's watching you. You might take a session or two before you kind of feel comfortable. (Participant 12, PCIT‐T)

##### 3.3.3.1 Unique Themes for the COS‐P GROUP

Similar to participants in the PCIT‐T group, those in the COS‐P group identified the clinician as an important treatment facilitator. In addition, they identified the group process and being around other parents as a positive. A number of participants who had attended the COS‐P group also discussed the ‘treatment journey’, with some noting that they initially did not know whether the treatment would be helpful but as they progressed through the program, they saw the benefit of it.

#### 3.3.4 Group Process

COS‐P participants also spoke positively about the group process, and in particular, they saw the benefit of listening to other mothers' experiences.

… hearing the other stories from the other mums… ‘oh yes and when I did this is what happened’ and ‘oh I tried doing this and this …. with the other mums you can kind of go ‘well okay their doing it right now’ so it's like ‘obviously they have that insight’. (Participant 13, COS‐P)

It's really good to share…. the other women sharing their experience. We learn from each other. It's really good. I like the group. (Participant 18, COS‐P)

#### 3.3.5 Treatment Journey

Some participants spoke about having taken some time to understand the program. As treatment progressed these mothers had “ah ha” moments (Participant 1) about how the sessions fit together and ways in which the program related to their lives.

… you sort of have like ‘ah ha’ moment throughout the class … [you] strung things together…and then when you can relate them to your own life. It may not have been that week but even a few weeks afterwards. (Participant 1, COS‐P)

I think it starts to make more sense around week three or four. A couple of weeks, it's all new concepts, which you haven't heard of before. But once you get to the middle of it, and you start connecting all the dots and it makes more sense. And then by the final…week, seven you're like, ‘Oh, yeah’. You see the whole picture. (Participant 15, COS‐P)

### 3.4 Theme 4: Treatment Barriers

Although participants in both the COS‐P and PCIT‐T groups reported benefiting from the programs they attended, this did not mean that attending and participating in the programs themselves were always easy processes. Participants from both groups discussed time commitment and distance to the treatment facility as treatment barriers.

I think just the length of time. Possibly, if it was even shortened into, maybe six sessions. Just, I feel like some of the content at the start could have been grouped, the first two weeks would be one week and get that all out of the way. And then move into the more deeper stuff. I feel it would use the time better, and make it all link better. (Participant 15, COS‐P)

It was a lot of, you know, going to appointments twice a week and then the daily homework. (Participant 21, PCIT‐T)

I suppose the only slight inconvenience is that you guys are in [suburb name] and I'm up this way so a little bit further away than everybody else. (Participant 1, COS‐P)

…used to take us three and a half hours out of the day and, you know, with [child] hating to travel that causes a certain amount of stress. (Participant 6, PCIT‐T)

In addition to time commitments and distance, some participants in the COS‐P group identified inconsistent attendance by other group members as a factor that impacted the treatment process.

There were so many different mums coming and going and at one stage there were about ten mums and then there were always three of us. Do you know what I mean? It was just annoying. The attendance, you either come or you don't. (Participant 20)

## 4 Discussion

Taken together, study results suggest that irrespective of whether they received PCIT‐T or COS‐P, parents felt that they experienced a range of positive gains in terms of toddler behavior, parental wellbeing, and relationships (including but not limited to, the parent‐toddler relationship). Importantly, participants in both groups identified the clinician as a key reason for the positive program outcomes and the time commitment as a significant constraint or barrier. Additional program‐specific facilitators included the group process and treatment journey (COS‐P group) and the live coaching and manualized protocol (PCIT‐T group).

Participants in both the COS‐P and PCIT‐T groups believed that the clinician was a key facilitator of change. Specifically, participants expressed their appreciation of the clinician's knowledge and competence, and in the PCIT‐T group, there was also a focus on feeling personally supported and feeling like they were not alone. This is important because it suggests that even though both programs are manualised, the skill of the clinician in creating a safe space and strong therapeutic alliance and delivering the program material in a way that meets the individual needs of parents, are paramount. These ideas are not new: similar themes emerged in the two previous qualitative studies of COS‐P and PCIT‐T (Kohlhoff, Cibralic, and Morgan 2020; Maxwell et al. 2021). Relationships—including that between the clinician and parent—are indeed central to attachment‐based parenting programs. The integral role of the clinician has also been emphasized in the wider literature about factors that facilitate success in parenting programs (Koerting et al. 2013) and adult psychotherapy (Horvath and Symonds 1991). Thus, while the important role of the clinician or therapist is not a new concept, it is a finding that aligns with broader literature, and which gives rise to clear implications for COS‐P and PCIT‐T clinical service provision. Specifically, to maximize outcomes from these programs, facilitators should be well‐skilled and competent/familiar with the program material and content. Importantly, they should be able to create a safe and welcoming environment, so that parents feel safe and supported to learn, reflect, and make changes in their lives. Within an attachment theory perspective, they must possess the capacity to provide a ‘safe haven’ and ‘secure base’ for the parent, so the parent can be vulnerable and feel supported, and explore new ideas and develop new ways of interacting with their child. This has implications for managers who may be hiring or choosing clinicians to provide PCIT‐T or facilitate COS‐P programs, but it also highlights the value of training, support, and high quality clinical supervision for staff (Reay et al. 2019). With good supports and structures in place to support clinicians, it is more likely that clinicians will deliver the programs with fidelity, and in a way that maximizes client outcomes.

For COS‐P participants, the ‘group process’ was highlighted as a factor that contributed to program outcomes. The perceived value of the COS‐P group process aligns with results recently reported by Maxwell et al. (2021) (“there's a group process that happens alongside the content”, 461). It also suggests that the COS‐P program, which is a parent‐only program, allows parents to engage in discussion with other parents and the facilitator away from their children and normal routines. COS‐P aims to change parenting behavior through changes in parental reflective abilities. The opportunity to engage in reflection, both individually and as a group, is likely to be key in this. Participants also commented about the COS‐P ‘treatment journey’ as an important part of the change process, with some participants speaking about a slow process of realization, and one participant pinpointing an exact moment in the program where the material suddenly made sense to them (an “ah‐ha” moment). Given COS‐P's focus on increasing parents' reflectiveness and their ability to identify the underlying or hidden beliefs and emotions that drive their parenting behavior (and the things in themselves or the child that trigger these beliefs/emotions; ‘shark music’), it is encouraging that parents in this study had this experience.

One of the factors that parents in the PCIT‐T group perceived to have contributed to positive program outcomes was the live coaching. Live coaching is a unique and defining feature of the PCIT‐T program, and something that clearly differentiates it from COS‐P, so it is of note that participants found this to be useful. Interestingly, while PCIT‐T and COS‐P share the common aim of improving outcomes for children through changes in parenting, the programs differ with respect to the mode of program delivery: COS‐P uses a group program format and seeks to change parenting representations through observation and reflection, whereas PCIT‐T works with individual parent–child dyads and provides in‐the‐moment coaching to bring about changes in parenting behavior. It is therefore of note that participants in both groups enjoyed the process that they went through and could see its benefits. PCIT‐T participants also spoke positively about the manualized program. This suggests that the PCIT‐T program materials and content are acceptable and liked by parents, and that they feel that the PCIT‐T protocol was able to be delivered in a way that was still nuanced and personable to ensure that their individual needs were met.

Participants in both groups identified the time commitment required for the interventions as a barrier. For both interventions, there was a total treatment time of approximately 16 h: for the COS‐P program, this was in 2‐h sessions delivered over an 8‐week period, and for the PCIT‐T program, this was 45‐min sessions delivered twice weekly over an 8‐week period. Some families also mentioned travel time, as the interventions were provided at a community‐based early parenting service located in the south‐western region of Sydney, Australia. This finding aligns with previous research showing that time burden/resource limitations are predictive of parenting program attrition (Dumas et al. 2007), and thus, it is an issue that clinical services must address. It also relates to the other barrier identified in this study, namely inconsistent attendance by other COS‐P attendees. Presumably, this presented challenges for group cohesion and dynamics. Offering COS‐P and PCIT‐T programs in accessible locations, and where possible, offering telehealth options, are likely to enhance acceptability and attendance rates.

### 4.1 Limitations and Strengths

The study had several limitations that must be acknowledged. First, while it provides insights into the outcomes, facilitators and barriers associated with the COS‐P and PCIT‐T programs in this setting (a community‐based parenting service in South‐Western Sydney), and for this client group (parents of children aged 14–24 months presenting with concerns about toddler behavior), the applicability to other settings and client groups is unknown. It is encouraging that results align in many ways with those of the previous qualitative studies of parents perceptions of COS‐P and PCIT‐T (Kohlhoff, Cibralic, and Morgan 2020; Maxwell et al. 2021) but further studies in other contexts and with different clients groups are warranted. Second, all the participants in this study were also taking part in a larger RCT evaluating the program and as such were randomly allocated to receive the COS‐P or PCIT‐T intervention. It is possible that this may have introduced some bias to the sample (e.g., client perceptions about time commitment as a barrier, given that they were not able to choose which treatment program they received). Third, there were unequal sample sizes for the COS‐P and PCIT‐T groups in this study (*n* = 10 for COS‐P and *n* = 19 for PCIT‐T), and this is most likely a reflection of the fact that there was a greater level of attrition among participants allocated to COS‐P compared to those allocated to PCIT‐T. The unequal sample sized may have skewed thematic representations. The smaller sample sizes of COS‐P participants means that fewer COS‐P voices contributed to the themes identified. This may have led to an overemphasis of PCIT‐T participant perspectives. Furthermore, this study only included participants who completed either PCIT‐T or COS‐P interventions, it is possible that different themes may have emerged if parents who did not complete the programs were included in the sample. It would be beneficial for future studies to gain qualitative feedback from parents who drop out of COS‐P and PCIT‐T prematurely, to explore contributing factors. Fourth, with regards to the study procedure, study participants were not provided with an opportunity to review their transcripts prior to analysis, and while great care was taken to transcribe the interviews verbatim, it is possible that there were transcription errors which may have impacted results. In addition, while multiple coders were used to ensure that a range of perspectives were brought into the interactive and iterative discussion that led to the development of the coding scheme, there were no calculations of coding reliability between coders and so it is possible that the coding scheme contained biases. Future studies could address these methodological limitations by providing participants with the option to review their transcripts prior to analysis, and by including a formal check of inter‐rater reliability. Fifth, while the study used an inductive thematic analysis approach (Braun and Clarke 2006), it is possible that an alternative approach such as framework, template, or matrix analysis, also may have been useful (Gale et al. 2013; Ritchie et al. 2013). Framework analysis, for example, provides the flexibility to catalogue multiple meanings under an overarching heading, which may have been useful way to express the unique subthemes for PCIT‐T and COS‐P. A final limitation relates again to potential bias among the coders. While none of the interviewers were involved in the provision of the clinical interventions, all the coders were clinicians or clinician‐researchers who worked at the clinical service at which the study was conducted and at an unconscious level this may have impacted coding. To address this risk, effort was made to allocate multiple coders to each transcript (all transcripts double coded and many triple coded), to include coders from a range of professional backgrounds, and to allocate transcripts to coders about the programs that they had not delivered and did not have training/expertise in. Future studies may, however, benefit from including coders who were from a different setting and thus were able to come to the transcripts with “fresh eyes”. Despite these limitations, the current study had several strengths including the fact that it was conducted in a real‐world clinical setting (community‐based child treatment clinic); its use of a culturally diverse sample; childcare was offered to COS‐P participants to improve accessibility; and interview questions were designed to elicit both positive and negative perspectives on the programs, ensuring that contradictory viewpoints were actively considered in the analysis.

### 4.2 Clinical and Research Implications

There are many clinical and research implications of these findings. First, the finding that the clinician was a key part of program success speaks broadly to the need for clinicians to be cognizant of the importance of the therapeutic relationship when delivering attachment‐based interventions to parents of young children. It also highlights the importance of recruiting COS‐P group facilitators and PCIT‐T clinicians who possess the skills to not only deliver the program content competently but also to develop a safe and nurturing group process and/or therapeutic relationship with the parent and child. To ensure that COS‐P and PCIT‐T clinicians are supported to do this, they should be provided with regular reflective clinical supervision. Practically, it may also be beneficial to implement client‐report therapy tools to evaluate and monitor session‐by‐session therapeutic group processes (for COS‐P) and the quality of clinician‐client relationships (for both COS‐P and PCIT‐T). Examples include the Scale To Assess the Therapeutic Relationship (McGuire‐Snieckus et al. 2007) and the Therapeutic Bond Scales (Saunders et al. 1989).

A second key implication relates to the finding that parents identified the time burden associated with attending the parenting programs as a significant barrier. Any effort that service providers can make to reduce the time requirements and constraints for parents is likely to maximize attendance and consumer satisfaction (e.g., providing programs in central locations to reduce travel time, minimizing administrative intake procedures, providing childcare for COS‐P groups, providing telehealth options were possible). Future studies could also investigate the impact of treatment ‘dose’ on client outcomes. It is possible, for example, that similar client outcomes could be attained with less than 16 h of PCIT‐T treatment, or shorter COS‐P sessions.

Third, the fact that COS‐P and PCIT‐T were both so well‐liked and perceived by clients to be beneficial in a range of areas (child behavior, parenting confidence, and the quality of the parent–child relationship) is of interest given the differences between the programs in terms of theoretical underpinning. That is, while both interventions are informed by attachment theory principles and, as such, share a common focus on enhancing parental attunement and sensitivity to child cues, PCIT‐T differs from COS‐P because it also draws on social learning theory and infuses a behavioral approach. Relatedly, there are also differences in the intervention methods used (COS‐P intervenes at the representational level, using video material and reflection through group discussion, whereas PCIT‐T works primarily at the behavioral level, using live coaching to support behavioral change) and in delivery format (COS‐P is delivered in a group format and PCIT‐T is delivered individually). Given the differences between the intervention models, future studies should examine predictors of satisfaction/parent‐reported outcomes for the two programs and explore the mechanisms of change. It would also be of benefit to investigate specific treatment components that enhance outcomes and parental satisfaction to inform wider theoretical understanding of attachment‐based interventions and so that improvements to the programs can be made.

Fourth, the study raises questions about the suitability and acceptability of the PCIT‐T and COS‐P programs for different client groups. While the feedback about the programs was generally very positive, there were parents who reported no change in some areas (e.g., child behavior, relationship quality). It is possible that PCIT‐T or COS‐P might be more suited to some families than others due to factors such as parental personality or attachment style, or the severity or nature of the child behavior concerns. It is also possible that features of the programs may have impacted acceptability for some groups. For example, the difference in session frequency (i.e., one session per week for COS‐P and twice weekly sessions for PCIT‐T) or delivery format (i.e., group‐based sessions for COS‐P and individual sessions for PCIT‐T) may have affected client perceptions about program acceptability and impacts. Regarding PCIT‐T, there was a sense that the intensity of twice‐weekly sessions with homework between sessions created barriers for some families. Previous research highlights accessibility and competing demands on parents' time and resources as a barrier to engagement in parenting programs (Mytton et al. 2014). For families in this study who lived a long distance from the treatment center, COS‐P may have been preferable due to sessions being once per week only, and for parents who did not like big groups or being separated from their child, PCIT‐T may have been preferable. These possibilities raise questions about whether the interventions could be modified or implemented in a way that better meets the needs of different groups. For example, reviewing the homework component of PCIT‐T or offering weekly sessions.

Relatedly, given that COS‐P is a group‐based and PCIT‐T is delivered individually, this study raises interesting questions about the advantages and disadvantages of these differing treatment modalities. For service providers, group‐based programs such as COS‐P offer the potential advantage of being cheaper and less resource‐ and time‐intensive, allowing services to reach more families (Charles et al. 2011; Lopez Garcia et al. 2021). In contrast, PCIT‐T is considerably more resource‐intensive, requiring specialized infrastructure (e.g., specialized time out rooms, rooms with one‐way mirrors) and more staff time due to its individually delivered format. Consequently, group‐based programs such as COS‐P may be more scalable to, and more accessible in, resource‐limited settings. Economic cost–benefit analysis was not, however, a focus of the current study and is a worthy direction for future research. Group‐based parenting programs may also offer opportunities for parents to build social connections and normalize parenting challenges (Bennett et al. 2013). It is of note that in this study, social connections and support were not a theme that emerged among parents who attended the COS‐P groups and so future research could explore this in a more focused manner. In terms of clinical effectiveness of group versus individual parenting programs, Mathijs et al. (2024) recently conducted a meta‐analysis of the outcomes of group and individual parenting programs and concluded that both group‐based and individual programs improve parents' child behavior management and parenting stress but only group‐based programs improve parents' depressive symptoms. While it is not possible from the current study to determine the relative impact of group versus individual delivery modalities, it is of note that irrespective of whether they received COS‐P or PCIT‐T, participants reported significant positive changes following the interventions, and participants in both groups identified the unique aspects of the program as being a key mechanism of change (i.e., the group process for COS‐P and the live coaching for PCIT‐T).

## 5 Conclusions

In sum, this study provides an in‐depth exploration of parent perspectives about participation in COS‐P and PCIT‐T, and as such, provides valuable information about user acceptability and facilitators/barriers to positive outcomes. Parents who attended the programs spoke of having experienced positive gains in toddler behavior, parental wellbeing, and relationships. The clinician was identified as a key reason for the positive program outcomes, and the time commitment was a major challenge. In addition, parents who attended the COS‐P group spoke of the benefits of the group process and treatment journey, and those who attended the PCIT‐T program appreciated the live coaching and manualized protocol. Results of this study are important because they highlight COS‐P and PCIT‐T as programs that are well‐liked and appreciated by families experiencing early toddler behavioral issues. To maximize the success of these programs, however, results suggest that service providers may wish to (1) invest in practical support and clinical supervision for COS‐P and PCIT‐T clinicians and (2) minimize time commitment for families. To further explore these issues, future research could explore the specific qualities of effective COS‐P and PCIT‐T clinicians and the feasibility and effectiveness of adapted briefer versions of COS‐P and PCIT‐T. Economic return‐on‐investment studies and further clinical outcome studies to compare clinical outcomes of the COS‐P and PCIT‐T programs would also be of use.

## Author Contributions

J.K. designed the study. J.K., S.C., and N.W. were involved in data collection. All authors (J.K., S.C., N.W., S.M., L.L., L.R., and S.M.) were involved in data analysis. J.K. takes full responsibility for the data, the analyses and interpretation and the conduct of the research, and has full access to all of the data; J.K. wrote the first draft of the manuscript and all authors contributed to the final version. All authors have read and approved the manuscript.

## Ethics Statement

The research project was approved by the South Western Sydney Local Health District Human Research Ethics Committee (project number HREC/18/LPOOL/72).

## Consent

All participants provided written, informed consent prior to taking part in this study.

## Conflicts of Interest

Jane Kohlhoff, Nancy Wallace, and Sue Morgan are co‐developers of the Parent–Child Interaction Therapy—Toddler program.

## Data Availability

The data that support the findings of this study are available from the corresponding author upon reasonable request.
